# Disruption of Intranasal GnRH Neuronal Migration Route into the Brain Induced by Proinflammatory Cytokine IL-6: Ex Vivo and In Vivo Rodent Models

**DOI:** 10.3390/ijms242115983

**Published:** 2023-11-05

**Authors:** Viktoria Sharova, Vasilina Ignatiuk, Marina Izvolskaia, Liudmila Zakharova

**Affiliations:** Koltzov Institute of Developmental Biology, Russian Academy of Sciences, Vavilov Street, 26, 119334 Moscow, Russia

**Keywords:** mouse fetus, olfactory placodes, GnRH neuron migration, IL-6, LPS, IL-6mAbs, IL-6mRAbs, IgG modulation

## Abstract

Maternal immune activation results in altered levels of cytokines in the maternal–fetal system, which has a negative impact on fetal development, including the gonadotropin-releasing hormone (GnRH) system, which is crucial for the reproduction. Suppression of GnRH–neuron migration may be associated with cytokine imbalances, and primarily with proinflammatory cytokine interleukin (IL)-6. This study aimed to determine the effects of IL-6 and monoclonal antibody to IL-6 or IL-6R or polyclonal IgG on the formation of migration route of GnRH–neurons in ex vivo and in vivo rodent models on day 11.5 of embryonic development. The increased level of IL-6 in mouse nasal explants suppressed peripherin-positive fiber outgrowth, while this led to an increase in the number of GnRH–neurons in the nose and olfactory bulbs and a decrease in their number in the fetal brain. This effect is likely to be realized via IL-6 receptors along the olfactory nerves. The suppressive effect of IL-6 was diminished by monoclonal antibodies to IL-6 or its receptors and by IgG.

## 1. Introduction

Growing evidence indicates that maternal immune activation (MIA) affects the health of offspring in immunological, metabolic, and neurological disorders [1,2]. Crucial stress factors for the appearance of various pathological conditions, including reproductive disorders, in the offspring are, mainly, viral and bacterial infections that induce inflammation, especially during the early stage of pregnancy [3,4,5]. Bacterial infections in pregnancy can induce developmental abnormalities of the fetus without an interruption of pregnancy, and infertility can become a serious problem in the adult life of men and women [6,7]. It has been experimentally proven that injection of lipopolysaccharide (LPS), a cell wall component of Gram-negative bacteria (*Escherichia coli*), in high doses to female mice between gestational days 14 and 18 triggered preterm birth and intrauterine fetal death [8]. LPS-induced inflammation triggers a cascade of synthesis and secretion of various proinflammatory cytokines, interleukin (IL)-1β, IL-6, tumor necrosis factor (TNFα), neuronal and other mediators, all of which constitute significant risk factors for the developing body [9,10,11,12].

According to our data, LPS exposure on embryonic day 12 (ED12) in maternal–fetal biological fluids increases the levels of IL-6, leukemia inhibitory factor (LIF), and monocyte chemotaxis factor (MCP-1) and induces reproductive disorders in rodent offspring [13,14]. The IL-6 level was highest compared to other proinflammatory cytokines. During pregnancy, maternal cytokines (e.g., IL-6 and IL-17a) suppress MIA-induced autism-like phenotypes in offspring [15,16].

A number of studies discuss possible mechanisms via which stress factors can disrupt neuronal migration processes [17]. The migration of neurons, including gonadotropin-releasing hormone (GnRH) neurons, which emerge from olfactory placode epithelium, starts in the prenatal period, and this process is crucial for the formation and further integration of the neuronal network. A disruption of the migration process results in dysfunction of the brain and reproductive axis [14]. Since cytokines transmit migrational signals to newly generated neurons, increased cytokine levels are likely to trigger misguidance of neurons in the fetal brain under MIA [18]. MIA also selectively alters the expression of specific genes that are involved in the regulation of migration, cell proliferation, and axon guidance in the fetal brain [19,20]. It was discovered that maternal elevated IL-6 concentration during pregnancy was associated with variations in the frontolimbic circuitry in the newborn, and this in turn correlated with the infant’s cognition at 12 months [21].

To date, experience is accumulating in the use of immunoglobulins (IgG) to suppress the negative effect of inflammation in the treatment of recurrent miscarriage [22] and during local neuroinflammation in experiments [23,24]. Studies are being carried out on the effectiveness and safety of using monoclonal antibodies target proinflammatory cytokines and their receptors; in particular, using recombinant humanized monoclonal antibodies to the IL-6 receptor (IL-6RmAbs) of Tocilizumab in the treatment of severe forms of the coronavirus infection [25].

The purpose of this study was to investigate the influence of LPS- and IL-6-induced systemic inflammation and the anti-inflammatory effects of polyclonal IgG and monoclonal antibodies to IL-6 and its receptor (IgG1) on the intranasal migration of GnRH neurons into the brain during the ontogenesis of rodent fetuses, using in vivo and ex vivo models. The effect of IL-6, administered to female mice on day 11.5 of pregnancy, on the migration of GnRH neurons was studied in fetal mice on ED 14.5 and ED 18.5. The role of IL-6 in the formation of the migration pathway of GnRH neurons into the brain was also studied in an organotypic culture of the olfactory placodes of 11.5-day-old mouse fetuses after co-culturing these placodes with IL-6mAbs and IL-6RmAbs for 5 days. In addition, we examined the effect of IL-6RmAbs (Tocilizumab) and polyclonal IgG, administered 40 min after LPS to a female rat on day 12 of pregnancy, on the intranasal migration of GnRH neurons in rat fetuses on ED17 and ED19.

## 2. Results

### 2.1. GnRH Neuron Distribution after IL-6 Prenatal Treatment in Mouse Fetuses

**Figure 1:**
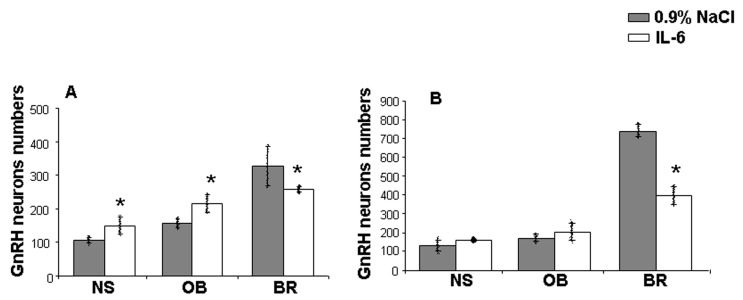
Distribution of GnRH neurons in different areas of their migration on ED14.5 (**A**) and ED18.5 (**B**) in mouse fetuses after the administration of saline (controls) or IL-6 (0.1 μg/kg, i.p.) on ED 11.5 (*n* = 20 fetuses from 2 control and 2 IL-6-treated pregnant mice for each ED). Mean ± SD. * *p* < 0.05. NS—nasal area; OB—olfactory bulb; BR—brain.

On day 11.5 of pregnancy, female mice were administered IL-6 at a dose (0.1 μg/kg) corresponding to its content in the blood of fetuses after prenatal exposure to LPS (45 μg/kg) [13]. On ED14.5, GnRH-immunoreactive (ir) neurons were located equally in all three regions studied: nasal area, region of the olfactory bulb, and brain area (Figure 1A). In the nasal area, they were detected on the olfactory and vomeronasal nerves, near the cribriform plate, and always migrating into the forebrain. The changes in the number of GnRH-ir neurons in the IL-6-treated group on ED 14.5 were significant across all the three identified areas of migration (Figure 1A). In the nasal and olfactory bulb areas, their number exceeded that of the control group (by about 38%). In the forebrain region, the number of GnRH-ir neurons decreased by 20% in the IL-6-treated group compared with the control group (Figure 1A).

**Figure 2:**
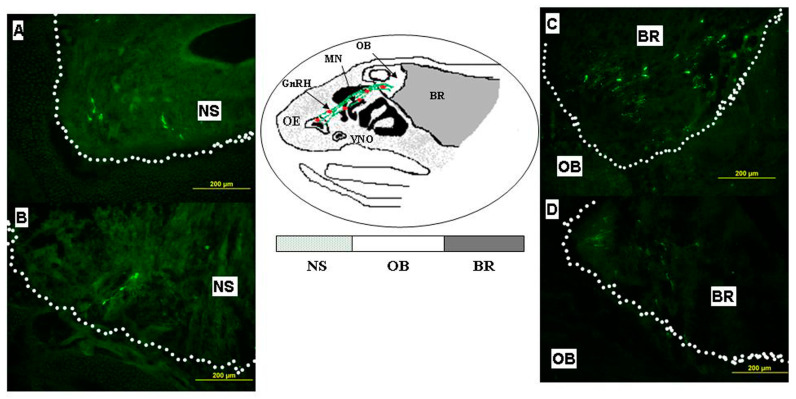
Schematic drawing of sagittal section of mouse head with GnRH neuron distribution. GnRH neuron distribution on ED14.5/ED18.5 in mouse fetuses after administration of IL-6 (**A**,**C**) or saline (**B**,**D**) in nasal (left side) and brain area (right side). Anti-GnRH immunostaining (green). The nasal and brain areas are marked with dots. Scale bar = 200 μm. BR—brain; GnRHn—gonadotropin-releasing hormone neurons; MN—nerves for GnRH neurons migration; NS—nasal area; OB—olfactory bulbs; OE—olfactory epithelium; VNO—vomeronasal organ.

On ED18.5, the number of GnRH-ir neurons increased compared with the number on ED14.5. The greatest number was observed in the forebrain area (Figure 1B). After the IL-6 treatment, a significant decrease (about 45%) in the total number was observed only in the forebrain region. In the nose and olfactory bulb areas, the total number of GnRH-ir neurons did not significantly increase in the IL-6 treated group (Figure 1B and Figure 2).

### 2.2. Effect of IL-6 on the Growth of Olfactory Nerve Fibers in an Organotypic Culture of Fetal Mouse Placodes

**Figure 3:**
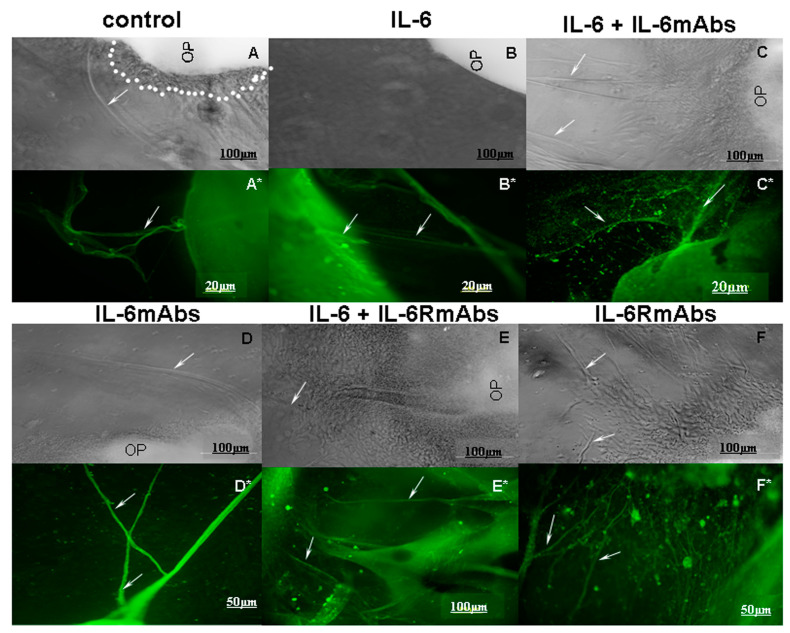
Images of ED11.5 mouse olfactory placodes after 5 days of cultivation (ED16.5): (**A**) with serum-free medium (control), (**B**) with IL-6 (10 ng/mL), (**C**) with IL-6 and IL-6mAbs (10 ng/mL each), (**D**) with IL-6mAbs (10 ng/mL), (**E**) with IL-6 and IL-6RmAbs (10 ng/mL each), and (**F**) with IL-6RmAbs (10 ng/mL). *n* = 20 placodes per group. After 5 days of cultivation, the olfactory placodes were stained for peripherin (1:50) in the same experimental groups (A*–F*). Peripherin-positive fibers (green) are marked with arrows. The halo with a layer of migrating fibroblasts is marked with dots. Scale bar: 20–100 μm. Abbreviation: NM—nasal mesenchyme, OP—olfactory pit.

**Figure 4:**
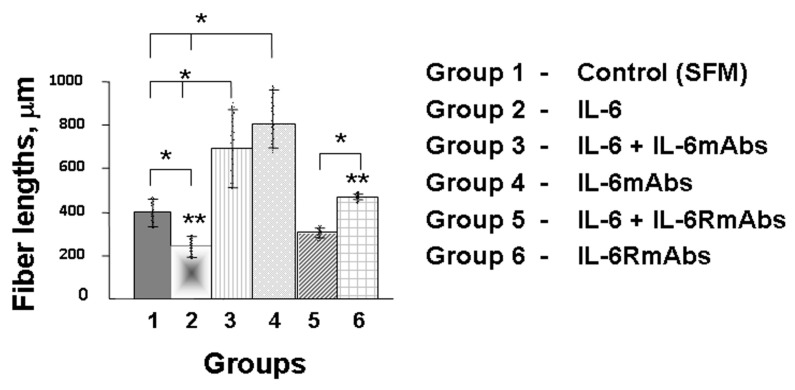
Graph showing the means of olfactory fiber lengths from nasal mesenchyme after 5 days of cultivation with composition of serum-free medium (control, group 1), with IL-6 (10 ng/mL, group 2), with IL-6 and IL-6mAbs (10 ng/mL each, group 3), with IL-6mAbs (10 ng/mL, group 4), with IL-6 and IL-6RmAbs (10 ng/mL each, group 5), and with IL-6RmAbs (10 ng/mL, group 6) (n ≥ 200 fibers per group). (*n* = 20 placodes per group). The data are expressed as mean ± SEM.; ANOVA; * *p* < 0.05 as compared with the control and IL-6 groups, ** *p* < 0.05 as compared with the IL-6 group.

**Figure 5:**
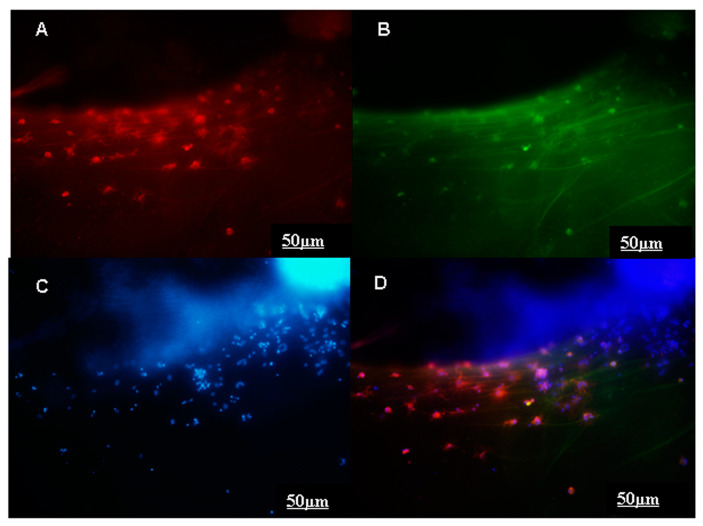
Images of migration of GnRH neurons on growing peripherin-positive axons after 7 days of cultivating ED11.5 mouse olfactory placodes. (**A**) Explants stained for GnRH (1:1000, red), (**B**) peripherin (1:50, green), (**C**) Hoechst 14533 (1:1000, blue): (**D**) overlay of red (**A**), green (**B**) and blue (**C**) filters. Scale bar: 50 μm.

Growing peripherin-positive axons were detected on the olfactory placodes of 11.5-day-old fetuses after 5 days of cultivation in a serum-free medium (ED16.5). In the control (group 1), the length of the axons reached about 400 µm (Figure 3A,A* and Figure 4). Optimally immunocytochemically stained GnRH neurons on growing peripherin-positive axons were identified after 7 days of placode cultivation (ED18.5) (Figure 5).

After 5 days of placode cultivation with IL-6 (10 ng/mL, group 2), a significant suppression of the growth of olfactory nerve fibers was observed (Figure 4). There were fewer fibers, and they were half as long compared with the control group (Figure 3B,B*). Group 2 also had stunted placodes, that is, placodes without visible axons or with short axons, as well as without the halo of a layer of migrating fibroblasts around the placode (Figure 3B), compared with the control (Figure 3A).

When IL-6 was co-cultivated with IL-6mAbs (10 ng/mL each, group 3), an increase in the length of axons was observed: this was twofold compared with the control and approximately fourfold compared with group 2, where placodes were cultivated only with IL-6 (Figure 4). At the same time, the axons were few in number (up to 2), but long compared with the control group, where up to 10 axons with varying degrees of length were recorded (Figure 3C,C*).

An increase in the growth of nerve axons was also observed when placodes were cultured with IL-6mAbs (IgG1) without IL-6 (group 4) (Figure 3D,D* and Figure 4).

In contrast, after co-cultivation of placodes with IL-6 and IL-6RmAbs (10 ng/mL each, group 5), axon growth increased only slightly and did not reach the length observed in the control group (Figure 4). At the same time, the axons had thick trunks and many branches (Figure 3E,E*). Cultivating placodes with IL-6RmAbs (IgG1) (group 6) without adding IL-6 also induced axon growth similar to that induced with IL-6mAbs (group 4) (Figure 3F,F*). However, the axons were half as long as in group 4 (Figure 4).

### 2.3. GnRH-ir Neuron Distribution after Prenatal LPS and IL-6RmAbs or IgG Treatment in Rat Fetuses

**Figure 6:**
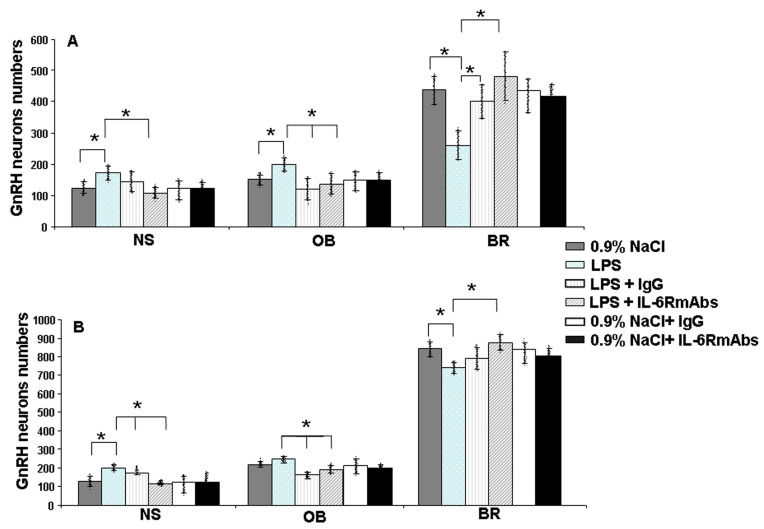
Distribution of GnRH neurons in different areas of their migration on ED17 (**A**) and ED19 (**B**) in rat fetuses after the administration of saline (controls), LPS (50 μg/kg, i.p.), LPS + IgG (1 mg/kg, i.v.), LPS + IL-6RmAbs (2 mg/kg, i.v.), saline + IgG (1 mg/kg, i.v.), or saline + IL-6RmAbs (2 mg/kg, i.v.) on ED11.5. IgG and IL-6RmAbs were administered i.v. 40 min after LPS i.p. (*n* = 20 fetuses from 2 control and 2 LPS-treated pregnant rats from each ED). Mean ± SD. * *p* < 0.05. NS—nasal area; OB—olfactory bulb; BR—brain.

After LPS administration to female rats on day 12 of pregnancy, the quantitative distribution of GnRH neurons across the three regions of their migration was similar to their distribution in mice treated with IL-6. On ED17, the number of GnRH neurons increased in the nasal and olfactory bulb regions (by 38% and 32%, respectively), and decreased in the forebrain region by 40% compared with the control group (Figure 6A). At a later time, on ED19, the number of GnRH neurons also increased in the rostral head (by 50% and 13%, respectively) and decreased by 11% in the anterior hypothalamus (Figure 6B).

Recombinant humanized IL-6RmAbs (IgG1, 2 mg/kg) or polyclonal IgG (1 mg/kg), administered to female rats 40 min after LPS, suppressed the suppressive effect of LPS on the migration of GnRH neurons in the nose and olfactory bulbs on ED17 and ED19 (Figure 6).

IL-6RmAbs restored the number of GnRH neurons by 80% in the forebrain region on ED 17 (Figure 6A) and by 20% in the anterior hypothalamus region on ED 19 (Figure 6B). Polyclonal IgG also suppressed the suppressive effect of LPS in the brain region. However, its effect was lower compared with that of IL-6RmAbs. The number of GnRH neurons increased by 50% on ED17 (Figure 6A) and by 10% on ED19 (Figure 6B) compared with the LPS-treated group (Figure 6).

The effect of specific antibodies to IL-6R was more pronounced than that of polyclonal IgG. After the administration of IL-6RmAbs, the number of GnRH neurons approached the control levels in all the studied areas and at all times.

Administration of IL-6RmAbs or IgG to LPS-untreated pregnant females did not result in significant changes in the numbers of GnRH neurons in nasal and olfactory bulb areas and in the fetal brain on ED17 and ED 19 (Figure 6).

## 3. Discussion

The research presented in this article is aimed at studying the influence of the proinflammatory cytokine IL-6 and the modulating effect of IgG class immunoglobulins on the intranasal migration of GnRH neurons into the brain during the ontogenesis of rodent fetuses.

To date, data have accumulated proving that perinatal infections largely determine neonatal and infant mortality rates or lead to maladaptation, and, in some cases, disability of children [11,26]. In experimental models, MIA via bacterial LPS results in altered levels of various cytokines in both mother and fetus [13]. Increased levels of cytokines induced via inflammation during the early stages of pregnancy can be one of the risk factors causing developmental disorders of the immune and neuroendocrine systems, including the hypothalamic–pituitary–gonadal axis (HPG axis), in fetuses [5,27]. The consequences of these disorders can develop throughout postnatal ontogenesis. Disruptions in HPG axis development may lead to sexual maturation problems and infertility [7,28].

A key part of the HPG axis, responsible for the reproductive health of sexually mature offspring, is the GnRH system. GnRH neurons emerge from the nasal placode during early embryonic development, ED10.5-ED11.5 in mice [29,30,31] and ED12.5-ED13 in rats [32,33,34], and migrate along olfactory axons until reaching the developing hypothalamus. Within the brain, GnRH neurons are found scattered from the olfactory bulbs to the hypothalamus, where the majority of neurons project their axons to the median eminence [30,35]. The initial stages in the development of GnRH neurons are likely to be their critical period, since LPS exposure on ED15 did not induce any significant changes in the total neuron number or in their distribution between migration areas in rat fetuses [13]. The development of GnRH neurons is regulated via various signaling molecules, including cytokines [36,37]. The cytokines synthesized by various cells of the body, LIF, MCP-1 and IL-6 in particular, take part in GnRH secretion, in the development of olfactory structures, and in the migration of GnRH neurons [3,5,38].

According to our previous results, LPS exposure on ED12 in maternal–fetal biological fluids increased the levels of proinflammatory cytokines: importantly; the IL-6 level was highest compared with other cytokines. Such LPS exposure also led to a suppression of GnRH migration and a delay in the formation of the necessary axonal connections [13,14]. We hypothesized that the suppression of GnRH neuron migration is associated with increased IL-6 levels. Our data indicate that the administration of IL-6 to female mice on day 11.5 of pregnancy, at a dose corresponding to its content in fetuses after exposure to LPS, causes a similar quantitative distribution of GnRH neurons in mouse fetuses on ED14.5 [13] (Figure 1A) and ED18.5 (Figure 1B); and in rats on ED17 and ED19 (Figure 6). An increase in the number of GnRH neurons was observed in the area of the nose and olfactory bulbs, and a decrease in the number of GnRH neurons was observed in the brain area compared with the group of animals not treated with IL-6. At birth there was no significant difference between the treated and control groups [13].

IL-6 is a cytokine with a unique pleiotropic function due to three major sites of signaling: the maternal immune system, the maternal/fetal interface (the placenta), and the fetal brain [39]. It has been suggested that IL-6 crosses the placenta barrier, leading to the loss of Purkinje cells in fetal hindbrain regions; indeed, the removal of IL-6 receptors in placental trophoblasts has prevented this phenotype [40]. Furthermore, IL-6 injection to fetal brains has led to disorders of synaptogenesis in glutamatergic neurons [41]. Increases in TNFa, IL-1, and IL-6 have been detected in the liver, brain, and plasma after a maternal LPS injection, but the gestational age and the injection timing need to be considered as impacting placental transport capacity [19,42].

The effect of IL-6 on GnRH neurons can be realized via specific IL-6 receptors, identified along the entire migratory path on the peripherin-positive olfactory and vomeronasal nerves of fetal mice from ED11.5 to ED14.5 [13]. We investigated the role of IL-6 in the formation of the migratory pathway of GnRH neurons into the brain in an organotypic culture of the olfactory placodes of 11.5-day-old mouse fetuses after co-cultivation with IL-6mAbs and IL-6RmAbs for 5 days. Nasal explants isolated on ED11.5 exhibit directed neuronal migration and axon growth [43]. After 3 days of cultivation (ED14.5), the nasal explants were symmetrical, with a layer of migrating fibroblasts in a halo and the first GnRH neurons starting to emerge [31]. Ex vivo GnRH neurons migrated along intermediate neurofilament peripherin-positive olfactory axons towards the olfactory bulb, and their migration ended on day 5 of cultivation [31]. Accordingly, the optimal length of axons for their further measurement ex vivo could be found on day 5 of cultivation (ED16.5). According to our data, increased levels of IL-6 suppressed fiber growth in an organotypic culture of fetal olfactory placodes (Figure 3B,B* and Figure 4). Additionally, there was no halo of migrating fibroblasts around the placodes (Figure 3B) compared with the control group (Figure 3A). IL-6mAbs reduced the effect of IL-6 (Figure 3C,C* and Figure 4). IL-6RmAbs increased axon growth in culture, but axon length did not reach the control levels and was shorter than when IL-6mAbs was added (Figure 3E,E* and Figure 4). This may be due to the small number of IL-6 receptors on short axons by day 5 of co-cultivation with IL-6 (ED16.5). Monoclonal antibodies (IgG1) also induced axon growth, but to a lesser extent than when they were co-cultivated with IL-6. We hypothesize that IgG1 may also perform a trophic function or stimulate the synthesis of regulatory growth factors in a serum-free culture of olfactory placodes (Figure 4).

Rat polyclonal IgG of unknown specificity and recombinant IL-6RmAbs (TCZ) administered to female rats on ED12 reduced the suppressive effect of LPS on GnRH neuron migration in the nasal area and fetal brain (Figure 6). The effect of immunoglobulins was most pronounced when the synthesis of proinflammatory cytokines induced via LPS had not yet reached its peak [13], that is, 40 min after LPS administration. IL-6mAbs (IgG1) administered to pregnant female mice immediately after the administration of double-stranded RNA (poly I:C) also normalized the induced abnormalities in adult offspring [39]. In experimental rodent models with LPS-induced endotoxemia accompanied by an increased level of proinflammatory cytokines, IgG and IgM suppressed the synthesis of these cytokines and the development of immunosuppression [44]. This process can be mediated via the binding of the IgG Fc fragment to the Fcγ receptor (FcγR) expressed on various cells, which modulates their functions and FcγR expression [45]. IgG can also have an anti-inflammatory effect, reducing cell sensitivity to signals via the toll-like receptors, which leads to a decrease in the synthesis of proinflammatory cytokines [24]. We supposed that the IgG effect is likely realized in two different ways: stimulation of olfactory axon growth or suppression of the IL-6 synthesis in a maternal–fetal system.

Attempts are currently being made to suppress the negative effects of proinflammatory cytokines with the help of intravenous IgG administration to animals and humans [23,24]. The efficacy and safety of monoclonal antibody administration against various proinflammatory cytokines and their receptors in the treatment of autoimmune and chronic inflammatory diseases have been assessed [46]. However, the use of cytokine-specific monoclonal antibodies during pregnancy requires special caution. Their approbation on experimental animals is necessary.

In line with these results, in vivo and ex vivo models have made it possible to identify one of the cytokines, specifically, IL-6, involved in the processes of intranasal migration of GnRH neurons.

## 4. Materials and Methods

### 4.1. Animals and Experimental Design (In Vivo Model)

Pregnant Balb/c mice initially weighing 18–20 g and Wistar rats weighing 200–250 g (Stolbovaya Breeding Center, Moscow, Russia) and their fetuses were used in this study. The animals were kept in standardized conditions (24 °C, 12:12 h light–dark cycle, food and water ad libitum). The pregnant females were anesthetized with pentobarbital (100 mg/kg body; Sanofi, Libourne, France) and the fetuses were removed from the uterus.

On day 11.5 of pregnancy (the day of conception was designated as ED0.5), the mouse females (*n* = 3 per group) were intraperitoneally (i.p.) injected with 100 µL of pyrogen-free 0.9% (control) or with IL-6 (Sigma Chemical Company, St. Louis, MO, USA, I-4767, 0.1 μg/kg body) in the same volume of saline (IL-6-treated group). The chosen IL-6 dose corresponds to its content in the fetal blood after LPS exposure on ED11.5 [13].

On day 12 of pregnancy (the day of conception was designated as ED1), the rat females (*n* = 3 per group) were i.p. injected with 500 μL pyrogen-free 0.9% saline or LPS *E. coli* (50 μg/kg body, i.p.) in 500 μL of saline (Sigma, L8274, USA). Forty minutes after LPS administration, when cytokine secretion had not reached its peak, the females were intravenously (i.v.) injected with either 0.2 mL of 0.9% saline, rat IgG (Sigma-Aldrich, Saint-Louis, MO, USA, 1 mg/kg) in 0.2 mL of saline, or recombinant humanized IL-6RmAbs (IgG1) (Tocilizumab, 2 mg/kg) (Chugai Pharma Manufacturing Co., Tokyo, Japan), optimal for rats and humans [47,48], in the same volume of saline. Doses of IgG and TCZ were chosen experimentally [14]. IL-6RmAbs and IgG were also injected into LPS-untreated animals (Figure 6).

### 4.2. GnRH Immunohistochemical Staining in Fetuses

Mouse fetuses (on ED14.5 or ED18.5) and rat fetuses (on ED17 or ED19) (*n* = 6 at each age) were used for GnRH immunohistochemical staining. The ED18.5 mouse fetuses or ED19 rat fetuses were perfused via the heart with 2–5 mL of 0.02 M phosphate-buffered saline (PBS, pH 7.4) and then with 2–3 mL 4% paraformaldehyde (PAF). For final fixation, the heads of fetuses from both age groups were fixed in 4% PAF in 0.1 M PBS (pH 7.4) for 3 h at room temperature, rinsed in 0.2 M PBS (pH 7.4) for 30 min, immersed in 25% sucrose in PBS for 36 h at 4 °C, and frozen in isopentane at −40 °C. The heads were stored at −80 °C before sectioning. Serial 12-μm-thick sagittal sections were prepared with a cryostat microtome (Leica, Nussloch, Germany) and mounted onto gelatine-coated slides.

The head sections were then incubated successively with: (1) 10% goat normal serum with 0.3% Triton X-100 (Sigma), for 30 min at room temperature, (2) primary rabbit anti-GnRH polyAbs (1: 3,000, Abcam, Boston, MA, USA) containing 1% bovine serum albumin (BSA) and 0.3% Triton X-100, for 48 h at 4 °C, (3) secondary Alexa-Fluor-488-conjugated goat anti-rabbit antibodies (1:500) (Abcam, 150077) with 1% BSA and 0.3% Triton X-100 in 0.02 M PBS at 4 °C for 2 h. Three macrohistological regions were chosen for the counting of GnRH-immunoreactive (ir) neurons: (1) the nasal head area, (2) the region of olfactory bulbs, and (3) the brain area (Figure 2). The sections were examined using a fluorescent microscope (Olympus DP70, Leica, Germany). For mono-immunostaining and image analysis, every second section in the whole series was used for counting the neurons, and the total value was doubled. The number of GnRH neurons was estimated using a 20× objective. GnRH-ir cell bodies with nuclei visible with a 40× objective were included in the count.

### 4.3. Preparation of Fetal Olfactory Mouse Placodes (Ex Vivo Model)

The effects of IL-6 and IL-6mAbs or IL-6mRAbs on olfactory fiber growth were studied in an ex vivo culture described by Klenke and Taylor-Burds [31]. Olfactory explants were prepared from embryos on ED11.5. For the preparation of explants, two lateral cuts going through the olfactory pit on either side of the nasal mesenchyme were made. Olfactory fibers extended from the bottom edge of the nasal mesenchyme. Nasal placodes were dissected from the developing nose and cut into butterfly shaped fragments and plated on glass cell culture slides (SPL Lifesciences, Republic of Korea) coated with 10 mL of chicken plasma (Ptitsefabrika Ptichnoe, Breeding Center, Moscow, Russia). Thrombin (10 mL; Sigma, St. Louis, MO, USA) was then added to the plasma/explant clot for further solidification. The olfactory explants were incubated in a defined serum-free medium (SFM, control) at 37 °C, 95:5 air:CO_2_, in a humidified incubator for 5 days [31,43].

In the experimental groups (*n* = 20 embryos per group), the olfactory explants were cultivated: (1) with composition of serum-free medium (control), (2) with IL-6 (RnD system, recombinant, 406-ML, 10 ng/mL), (3) with IL-6 and IL-6mAbs (RnD system, recombinant, MAB-406, clone MP520F3, 10 ng/mL each), (4) with IL-6mAbs (10 ng/mL), (5) with IL-6 and IL-6RmAbs (RnD system, recombinant, MAB-18301, clone 255820, 10 ng/mL each), and (6) with IL-6RmAbs (10 ng/mL). The IL-6 dose was chosen based on previously published data [13]. The treatment with IL-6 or/and IL-6mAbs (IL-6RmAbs) was initiated on day 1 of cultivation, before the growth of olfactory nerves outside of olfactory pits, and was replenished on day 3 of cultivation. The control cultures were maintained in SFM that was changed on day 3 of cultivation, as was done in the treatment groups. The attachment and growth of explants were controlled on each day of cultivation and microphotographs (4× objective) of individual explants were performed using the microscope Olympus IX51 (Olympus Corporation, Center Valley, PA, USA) and its affiliated image management software (DP Conroller, Olympus Optical Co., Ltd., Tokyo, Japan). SFMs were changed once on day 2 of cultivation. On day 5, the cultures were processed for immunocytochemistry. The general growth of olfactory axons was monitored using peripherin, which lines the migration path of GnRH neurons along the olfactory axons. Placode growth measurements were performed under a fluorescence microscope using the Topview software v4.1. In separate experiments, on day seven of placode cultivation, the presence of GnRH neurons along the fibers was assessed using immunocytochemical staining.

#### 4.3.1. Immunohistochemistry

For day 5 of cultivation onwards, the olfactory explants were fixed in 4% PAF, rinsed in PBS, and blocked by being incubated in 10% normal goat serum (NGS) and 0.3% Triton X-100 (Sigma, USA). After two PBS washes, the cultures were incubated with: (1) primary antibodies to chicken polyclonal peripherin (Abcam, 39374) (1:50), overnight at 4 °C in 1% BSA, (2) 0.3% Triton X-100, 3 times for 10 min at room temperature, (3) secondary goat anti-chicken Alexa-Fluor-488-conjugated antibodies (1:2000) (Abcam, 150169) with 1% NGS and 0.3% Triton X-100, at room temperature for 2 h, and (4) 0.02 M PBS, 3 times for 10 min. As a negative control, we omitted the primary antibodies of a particular result in the absence of immunostaining.

To determine the presence of GnRH neurons on peripherin-positive fibers, the explants were fixed in PAF on day 7 of cultivation and stained for GnRH, peripherin, and Hoechst. After two PBS washes, the cultures were incubated with: (1) primary rabbit anti-GnRH polyAbs (1: 3000, Abcam) and chicken polyclonal peripherin (Abcam, 39374) (1:50) overnight at 4° C in 1% BSA; (2) 0.3% Triton X-100 3 times for 10 min at room temperature, (3) secondary donkey anti-rabbit Alexa-Fluor-568-conjugated to GnRH (1:2000) (Abcam, 175470) and goat anti-chicken Alexa-Fluor-488-conjugated to peripherin (1:2000) (Abcam, 150169) with 1% NGS and 0.3% Triton X-100, at room temperature for 2 h; (4) 0.02 M PBS, 3 times for 10 min; (5) Hoechst (Sigma, 14533) (1:1000) in PBS, for 2 min at room temperature; and (6) 0.02 M PBS, 3 times for 10 min.

#### 4.3.2. The Measurement of Olfactory Fiber Growth

The measurement was performed under a fluorescent Keyence BZ-9000E microscope (Itasca, IL, USA) connected to a video camera (CCD) and a computer (IBM PC Pentium 60, Boca Raton, FL, USA). The images of each explant with peripherin-positive fibers were captured in the area of 0.5 mm from the edge of the explant. The images were received using the affiliated image management software (BZ-II Analizer, Osaka, Japan). The images were analyzed using the ToupView software (ToupTek Photonics, Hangzhou, China). Because no specific marker exists for the axons on which GnRH neurons migrate from the olfactory epithelium, the overall olfactory axon growth was monitored using the intermediate neurofilament peripherin. Since according to previously published data [13,43], all the olfactory axons along which GnRH neurons migrate from the olfactory pit to the forebrain are positive for peripherin and for the IL-6 receptor, the length of all peripherin-positive axons migrating from the olfactory pit to the cellular surface covering the culture cell on the slide was measured. For fiber measurements, the outermost zone containing the peripherin fiber network was recorded and the length of all peripherin-positive fibers was measured in each culture dish. The starting point was the beginning of the fiber from the placode, the end point was the visually visible end of the fiber. After that, the peripherin-positive fibers were defined in the ToupView software (ToupTek Photonics, Hangzhou, China), the data were converted to grayscale mode, and the fiber length of each explant was determined by defining the tracing connectivity. The length of each peripherin-positive fiber in µm was measured using ToupView software (ToupTek Photonics, Hangzhou, China), as described earlier [49], on the cells migrating from the olfactory pit in each fluorescent image obtained in the explants of all groups studied (n ≥ 200 fibers from different explants for each group).

### 4.4. Statistical Analysis

The results are expressed as mean ± SEM for each group. All data sets met the assumption of a normal distribution and homogeneity of variance. The statistical analysis of the results was performed using a one-way ANOVA test. For the entire statistical analysis: * *p* < 0.05, ns (not significant).

## 5. Conclusions

The data obtained indicate that one of the mediators involved in the processes of intranasal migration of GnRH neurons into the brain is IL-6. Following exposure to IL-6 on ED11.5, which is critical for the development of the GnRH system in mice, the number of GnRH neurons increased in the nasal and olfactory bulb regions and decreased in the forebrain region, similar to the effect of LPS on ED14.5 and ED18.5. After 5 days of cultivating the olfactory placodes of 11.5-day-old mouse fetuses ex vivo with IL-6, the growth of olfactory nerve fibers, which are a migratory pathway for GnRH neurons, was suppressed. IL-6 monoclonal antibodies reduced the suppressive effect of IL-6. Forty minutes after exposure to LPS, when the synthesis of proinflammatory cytokines, including IL-6, had not yet reached its peak, blockade of the IL-6 receptor with recombinant IL-6RmAbs reduced the negative effects of LPS in rat fetuses, indicating the participation of the cytokine in these processes. Polyclonal IgG, although non-specific, also modulated inflammatory processes.

## Data Availability

Data available on request due to restrictions e.g., privacy or ethical. The data presented in this study are available on request from the corresponding author.

## Abbreviations

BR	brain
BSA	bovine serum albumin
ED	day of embryonic development (embryonic day)
GnRH	gonadotropin releasing hormone
HPG	hypothalamic–pituitary–gonadal (axis)
IgG	immunoglobulin G
IL	interleukin
IL-6mAbs	monoclonal anti-interleukin 6 antibodies
IL-6RmAbs	monoclonal anti-interleukin 6 receptor antibodies
ir	immunoreactive
LIF	leukemia inhibitory factor
LPS	lipopolysaccharide
MCP-1	monocyte chemotactic protein 1
MIA	maternal immune activation
NGS	normal goat serum
NS	nasal area
OB	olfactory bulbs
OE	olfactory epithelium
PAF	paraformaldehyde
PBS	phosphate buffered saline
PolyAbs	polyclonal antibodies
SFM	serum-free medium
TNFα	tumor necrosis factor α
VNO	vomeronasal organ

## References

[1] Arck P.C., Hecher K. (2013). Fetomaternal immune cross-talk and its consequences for maternal and offspring’s health. Nat. Med..

[2] Estes M.L., Prendergast K., MacMahon J.A., Cameron S., Aboubechara J.P., Farrelly K., Sell G.L., Haapanen L., Schauer J.D., Horta A. (2020). Baseline immunoreactivity before pregnancy and poly(I:C) dose combine to dictate susceptibility and resilience of offspring to maternal immune activation. Brain Behav. Immun..

[3] Wu S., Wolfe A. (2012). Signaling of cytokines is important in regulation of GnRH neurons. Mol. Neurobiol..

[4] Wang H.L., Pei D.E., Yang R.D., Wan C.L., Ye Y.M., Peng S.S., Zeng Q.Q., Yu Y. (2019). Prenatal maternal vaginal inflammation increases anxiety and alters HPA axis signalling in adult male mice. Int. J. Dev. Neurosci..

[5] Zakharova L., Sharova V., Izvolskaia M. (2020). Mechanisms of Reciprocal Regulation of Gonadotropin-Releasing Hormone (GnRH)-Producing and Immune Systems: The Role of GnRH, Cytokines and Their Receptors in Early Ontogenesis in Normal and Pathological Conditions. Int. J. Mol. Sci..

[6] Wang H., Yang L.L., Hu Y.F., Wang B.W., Huang Y.Y., Zhang C., Chen Y.H., Xu D.X. (2014). Maternal LPS exposure during pregnancy impairs testicular development, steroidogenesis and spermatogenesis in male offspring. PLoS ONE.

[7] Izvolskaia M., Ignatiuk V., Ismailova A., Sharova V., Zakharova L. (2021). IgG modulation in male mice with reproductive failure after prenatal inflammation. Reproduction.

[8] Hudalla H., Karenberg K., Kuon R.J., Pöschl J., Tschada R., Frommhold D. (2018). LPS-induced maternal inflammation promotes fetal leukocyte recruitment and prenatal organ infiltration in mice. Pediatr. Res..

[9] Bernardi M.M., Kirsten T.B., Matsuoka S.M., Teodorov E., Habr S.F., Penteado S.H., Palermo-Neto J. (2010). Prenatal lipopolysaccharide exposure affects maternal behavior and male offspring sexual behavior in adulthood. Neuroimmunomodulation.

[10] Rose D.R., Careaga M., Van de Water J., McAllister K., Bauman M.D., Ashwood P. (2017). Long-term altered immune responses following fetal priming in a non-human primate model of maternal immune activation. Brain Behav. Immun..

[11] Izvolskaia M., Sharova V., Zakharova L. (2020). Perinatal Inflammation Reprograms Neuroendocrine, Immune, and Reproductive Functions: Profile of Cytokine Biomarkers. Inflammation.

[12] Yasumatsu K., Nagao J.I., Arita-Morioka K.I., Narita Y., Tasaki S., Toyoda K., Ito S., Kido H., Tanaka Y. (2020). Bacterial-induced maternal interleukin-17A pathway promotes autistic-like behaviors in mouse offspring. Exp. Anim..

[13] Sharova V.S., Izvolskaia M.S., Zakharova L.A. (2015). Lipopolysaccharide-induced maternal inflammation affects the gonadotropin-releasing hormone neuron development in fetal mice. Neuroimmunomodulation.

[14] Ignatiuk V., Izvolskaia M., Sharova V., Zakharova L. (2023). Disruptions in Hypothalamic-Pituitary-Gonadal Axis Development and Their IgG Modulation after Prenatal Systemic Inflammation in Male Rats. Int. J. Mol. Sci..

[15] Hsiao E.Y., Patterson P.H. (2011). Activation of the maternal immune system induces endocrine changes in the placenta via IL-6. Brain Behav. Immun..

[16] Choi G.B., Yim Y.S., Wong H., Kim S., Kim H., Kim S.V., Hoeffer C.A., Littman D.R., Huh J.R. (2016). The maternal interleukin-17a pathway in mice promotes autism-like phenotypes in offspring. Science.

[17] Hwang H.M., Ku R.Y., Hashimoto-Torii K. (2019). Prenatal Environment That Affects Neuronal Migration. Front. Cell Dev. Biol..

[18] Du M.R., Wang S.C., Li D.J. (2014). The integrative roles of chemokines at the maternal-fetal interface in early pregnancy. Cell Mol. Immunol..

[19] Oskvig D.B., Elkahloun A.G., Johnson K.R., Phillips T.M., Herkenham M. (2012). Maternal immune activation by LPS selectively alters specific gene expression profiles of interneuron migration and oxidative stress in the fetus without triggering a fetal immune response. Brain Behav. Immun..

[20] Lombardo M.V., Moon H.M., Su J., Palmer T.D., Courchesne E., Pramparo T. (2018). Maternal immune activation dysregulation of the fetal brain transcriptome and relevance to the pathophysiology of autism spectrum disorder. Mol. Psychiatry.

[21] Rasmussen J.M., Graham A.M., Entringer S., Gilmore J.H., Styner M., Fair D.A., Wadhwa P.D., Buss C. (2019). Maternal Interleukin-6 concentration during pregnancy is associated with variation in frontolimbic white matter and cognitive development in early life. Neuroimage.

[22] Han A.R., Lee S.K. (2018). Immune modulation of i.v. immunoglobulin in women with reproductive failure. Reprod. Med. Biol..

[23] Domínguez-Soto Á., Simón-Fuentes M., de Las Casas-Engel M., Cuevas V.D., López-Bravo M., Domínguez-Andrés J., Saz-Leal P., Sancho D., Ardavín C., Ochoa-Grullón J. (2018). IVIg Promote Cross-Tolerance against Inflammatory Stimuli In Vitro and In Vivo. J. Immunol..

[24] Sawa T., Kinoshita M., Inoue K., Ohara J., Moriyama K. (2019). Immunoglobulin for Treating Bacterial Infections: One More Mechanism of Action. Antibodies.

[25] Berardicurti O., Ruscitti P., Ursini F., D’Andrea S., Ciaffi J., Meliconi R., Iagnocco A., Cipriani P., Giacomelli R. (2020). Mortality in tocilizumab-treated patients with COVID-19: A systematic review and meta-analysis. Clin. Exp. Rheumatol..

[26] Noyola D.E., Demmler G.J., Nelson C.T., Griesser C., Williamson W.D., Atkins J.T., Rozelle J., Turcich M., Llorente A.M., Sellers-Vinson S. (2001). Houston Congenital CMV Longitudinal Study Group. Early predictors of neurodevelopmental outcome in symptomatic congenital cytomegalovirus infection. J. Pediatr..

[27] Ignatiuk V.M., Izvolskaya M.S., Sharova V.S., Voronova S.N., Zakharova L.A. (2019). Disruptions in the reproductive system of female rats after prenatal lipopolysaccharide-induced immunological stress: Role of sex steroids. Stress.

[28] Barabás K., Szabó-Meleg E., Ábrahám I.M. (2020). Effect of Inflammation on Female Gonadotropin-Releasing Hormone (GnRH) Neurons: Mechanisms and Consequences. Int. J. Mol. Sci..

[29] Schwanzel-Fukuda M., Pfaff D.W. (1989). Origin of luteinizing hormone-releasing hormone neurons. Nature.

[30] Wray S. (2010). From nose to brain: Development of gonadotrophin-releasing hormone-1 neurones. J. Neuroendocrinol..

[31] Klenke U., Taylor-Burds C. (2012). Culturing embryonic nasal explants for developmental and physiological study. Curr. Protoc. Neurosci..

[32] Daikoku-Ishido H., Okamura Y., Yanaihara N., Daikoku S. (1990). Development of the hypothalamic luteinizing hormone-releasing hormone-containing neuron system in the rat: In vivo and in transplantation studies. Dev. Biol..

[33] Yoshida K., Tobet S.A., Crandall J.E., Jimenez T.P., Schwarting G.A. (1995). The migration of luteinizing hormone-releasing hormone neurons in the developing rat is associated with a transient, caudal projection of the vomeronasal nerve. J. Neurosci..

[34] Schwanzel-Fukuda M. (1999). Origin and migration of luteinizing hormone-releasing hormone neurons in mammals. Microsc. Res. Tech..

[35] Hoffman G.E., Finch C.E. (1986). LHRH neurons in the female C57BL/6J mouse brain during reproductive aging: No loss up to middle age. Neurobiol. Aging.

[36] Oleari R., Massa V., Cariboni A., Lettieri A. (2021). The Differential Roles for Neurodevelopmental and Neuroendocrine Genes in Shaping GnRH Neuron Physiology and Deficiency. Int. J. Mol. Sci..

[37] Chachlaki K., Oleari R. (2023). Editorial: Physiological and pathological aspects of GnRH neuron system development. Front. Endocrinol..

[38] Bornstein S.R., Rutkowski H., Vrezas I. (2004). Cytokines and steroidogenesis. Mol. Cell Endocrinol..

[39] Smith S.E., Li J., Garbett K., Mirnics K., Patterson P.H. (2007). Maternal immune activation alters fetal brain development through interleukin-6. J. Neurosci..

[40] Wu W.L., Hsiao E.Y., Yan Z., Mazmanian S.K., Patterson P.H. (2017). The placental interleukin-6 signaling controls fetal brain development and behavior. Brain Behav. Immun..

[41] Mirabella F., Desiato G., Mancinelli S., Fossati G., Rasile M., Morini R., Markicevic M., Grimm C., Amegandjin C., Termanini A. (2021). Prenatal interleukin 6 elevation increases glutamatergic synapse density and disrupts hippocampal connectivity in offspring. Immunity.

[42] Ashdown H., Dumont Y., Ng M., Poole S., Boksa P., Luheshi G.N. (2006). The role of cytokines in mediating effects of prenatal infection on the fetus: Implications for schizophrenia. Mol. Psychiatry.

[43] Fueshko S., Wray S. (1994). LHRH cells migrate on peripherin fibers in embryonic olfactory explant cultures: An in vitro model for neurophilic neuronal migration. Dev. Biol..

[44] Kyvelidou C., Sotiriou D., Zerva I., Athanassakis I. (2018). Protection Against Lipopolysaccharide-Induced Immunosuppression by IgG and IgM. Shock.

[45] Chaigne B., Mouthon L. (2017). Mechanisms of action of intravenous immunoglobulin. Transfus. Apher. Sci..

[46] Sheppard M., Laskou F., Stapleton P.P., Hadavi S., Dasgupta B. (2017). Tocilizumab (Actemra). Hum. Vaccin. Immunother..

[47] Wakabayashi A., Sawada K., Nakayama M., Toda A., Kimoto A., Mabuchi S., Kinose Y., Nakamura K., Takahashi K., Kurachi H. (2013). Targeting interleukin-6 receptor inhibits preterm delivery induced by inflammation. Mol. Hum. Reprod..

[48] Ding L., Bai C., Liu Y. (2018). Interleukin-6 contributes to myocardial damage in pregnant rats with reduced uterine perfusion pressure. Braz. J. Med. Biol. Res..

[49] Tzekova N., Heinen A., Bunk S., Hermann C., Hartung H.P., Reipert B., Küry P. (2015). Immunoglobulins stimulate cultured Schwann cell maturation and promote their potential to induce axonal outgrowth. J. Neuroinflamm..

